# DC32, a Dihydroartemisinin Derivative, Ameliorates Collagen-Induced Arthritis Through an Nrf2-p62-Keap1 Feedback Loop

**DOI:** 10.3389/fimmu.2018.02762

**Published:** 2018-11-27

**Authors:** Menglin Fan, Yanan Li, Chunhua Yao, Xiufeng Liu, Jihua Liu, Boyang Yu

**Affiliations:** State Key Laboratory of Natural Products, Jiangsu Key Laboratory of TCM Evaluation and Translational Research, School of Traditional Chinese Pharmacy, China Pharmaceutical University, Nanjing, China

**Keywords:** DC32, rheumatoid arthritis (RA), collagen-Induced arthritis (CIA), Nrf2-p62-Keap1 feedback loop, Keap1/Nrf2/HO-1 pathway

## Abstract

Artemisinins have been reported to have diverse functions, such as antimalaria, anticancer, anti-inflammation, and immunoregulation activities. DC32 [(9α,12α-dihydroartemisinyl) bis(2′-chlorocinnmate)], a dihydroartemisinin derivative possessing potent immunosuppressive properties, was synthesized in our previous study. Collagen-induced arthritis (CIA) in DBA/1 mice and inflammatory model in NIH-3T3 cells were established to evaluate the effect of DC32 on RA and discover the underlying mechanisms. The results showed that DC32 could markedly alleviate footpad inflammation, reduce cartilage degradation, activate the Nrf2/HO-1 signaling pathway, and increase the transcription of p62 in DBA/1 mice with CIA. Further mechanistic exploration with NIH-3T3 cells indicated that DC32 could increase the transcription, expression, and nuclear translocation of Nrf2. In addition, DC32 promoted degradation of Keap1 protein and upregulated HO-1 and p62 expression. Furthermore, the effect of DC32 on Keap1 degradation could be prevented by p62 knockdown using siRNA. Administration of DC32 could inhibit the activation of Akt/mTOR and ERK, and pretreatment of NIH-3T3 cells with the autophagy inhibitor 3-methyladenine (3-MA) attenuated the degradation of Keap1 induced by DC32. These results suggest that DC32 inhibits the degradation of Nrf2 by promoting p62-mediated selective autophagy and that p62 upregulation contributed to a positive feedback loop for persistent activation of Nrf2. In summary, our present study demonstrated that DC32 significantly suppressed rheumatoid arthritis (RA) via the Nrf2-p62-Keap1 feedback loop by increasing the mRNA and protein levels of Nrf2 and inducing p62 expression. These findings provide new mechanisms for artemisinins in RA treatment and a potential strategy for discovering antirheumatic drugs.

## Introduction

Rheumatoid arthritis (RA) is one of the most prevalent autoimmune and degenerative joint diseases with immune hyperactivation and synovitis (1, 2). Non-steroidal anti-inflammatory drugs (NSAIDs), disease-modifying antirheumatic drugs (DMARDs), and glucocorticoids are the most commonly used drugs for the clinical treatment of RA (3). Biological DMARDs, such as TNF inhibitors and IL-6 receptor inhibitors, are now used worldwide for the treatment of RA. However, these biological DMARDs fail to control disease progression in all patients. Moreover, most of these drugs have side effects, such as infection and renal toxicity, during long-term usage (4, 5). Thus, there is still an urgent need for new therapeutic options for RA.

NF-E2-related factor 2 (Nrf2) is a nuclear transcription factor that regulates the expression of many crucial antioxidant and anti-inflammatory genes. The Nrf2 signaling pathway is negatively regulated by Kelch-like ECH associating protein 1 (Keap1). Nrf2 interacts with Keap1 in the cytoplasm and is rapidly degraded by the ubiquitin-proteasome pathway under normal conditions (6). Signals from oxidative stresses or electrophilic insults target the Nrf2-Keap1 complex and dissociate Keap1 from Nrf2, which then translocates to the nucleus and enhances the transcription of enzymes such as heme oxygenase-1 (HO-1) and NAD(P)H-quinone oxidoreductase 1 (NQO1) (7). Recent reports indicate that the Nrf2-mediated antioxidant pathway can be activated by natural and synthetic chemical substances that induce the transcription, expression and nuclear translocation of Nrf2 and facilitate the degradation of Keap1 (8–10). Keap1 is mainly degraded through the ubiquitin-proteasome pathway and autophagy-lysosome pathway (11). Lysosomal degradation of Keap1 is mediated by p62/SQSTM1 (p62), a stress-inducible adaptor protein involved in selective autophagy (12, 13). In addition to competing with Nrf2 for the Keap1 binding site and sequestering Keap1 into aggregates, p62 also brings Keap1 to autolysosomes for degradation in selective autophagy. The Nrf2-Keap1 interaction, which is blocked by the accumulation of p62 and Nrf2, is increased in the cytoplasm, resulting in activation of the Nrf2/HO-1 pathway (12, 14–16). In addition, *p62/SQSTM1* is also a target gene of Nrf2, and its expression can be induced by Nrf2 under oxidative stress conditions, further facilitating the activation of Nrf2 by blocking its degradation (17).

It was reported that activation of Nrf2/HO-1 signaling plays a critical role in the prevention and relief of RA. Nrf2 is the transcription factor of HO-1, which is considered a crucial cytoprotective protein. Induction of HO-1 expression protects against cartilage erosion and decreases the secretion of proinflammatory cytokines in the collagen-induced arthritis (CIA) model (18). Nrf2 knockout significantly aggravates cartilage destruction and accelerates the effector phase of arthritis in mice (19, 20), and upregulating the expression of Nrf2 exerts anti-inflammatory effects in RA (21). Therefore, activation of Nrf2 is a possible therapeutic mechanism for discovering new drugs for treating RA and other autoimmune diseases.

Artemisinin and its derivatives are widely used antimalaria drugs with antiviral, anti-inflammatory, anticancer, and immunosuppressive activities. Given the safety and these many beneficial properties of artemisinins, many studies have been carried out to investigate and develop their potential for treating immune diseases (22–26). Some evidence has suggested that the Nrf2-Keap1 pathway plays critical roles in the anti-inflammatory activity of artemisinins. It was reported that artesunate could protect against septic lung injury through activation of Nrf2/HO-1 signaling (27). Furthermore, the resistance of artesunate-induced ferroptosis in cancer cells and the antineuroinflammatory properties of artemether have both been connected to the activation of Nrf2 (28, 29), suggesting the ability of artemisinins to activate Nrf2. Silencing of Keap1 decreased artesunate sensitivity in cancer cells, indicating a close relationship between artesunate and Keap1 (28). The ability to activate the Nrf2/HO-1 pathway is likely involved in the anti-inflammation properties and immune regulation of artemisinins, but the mechanism of Keap1/Nrf2/HO-1 activation remains elusive.

**Figure 1:**
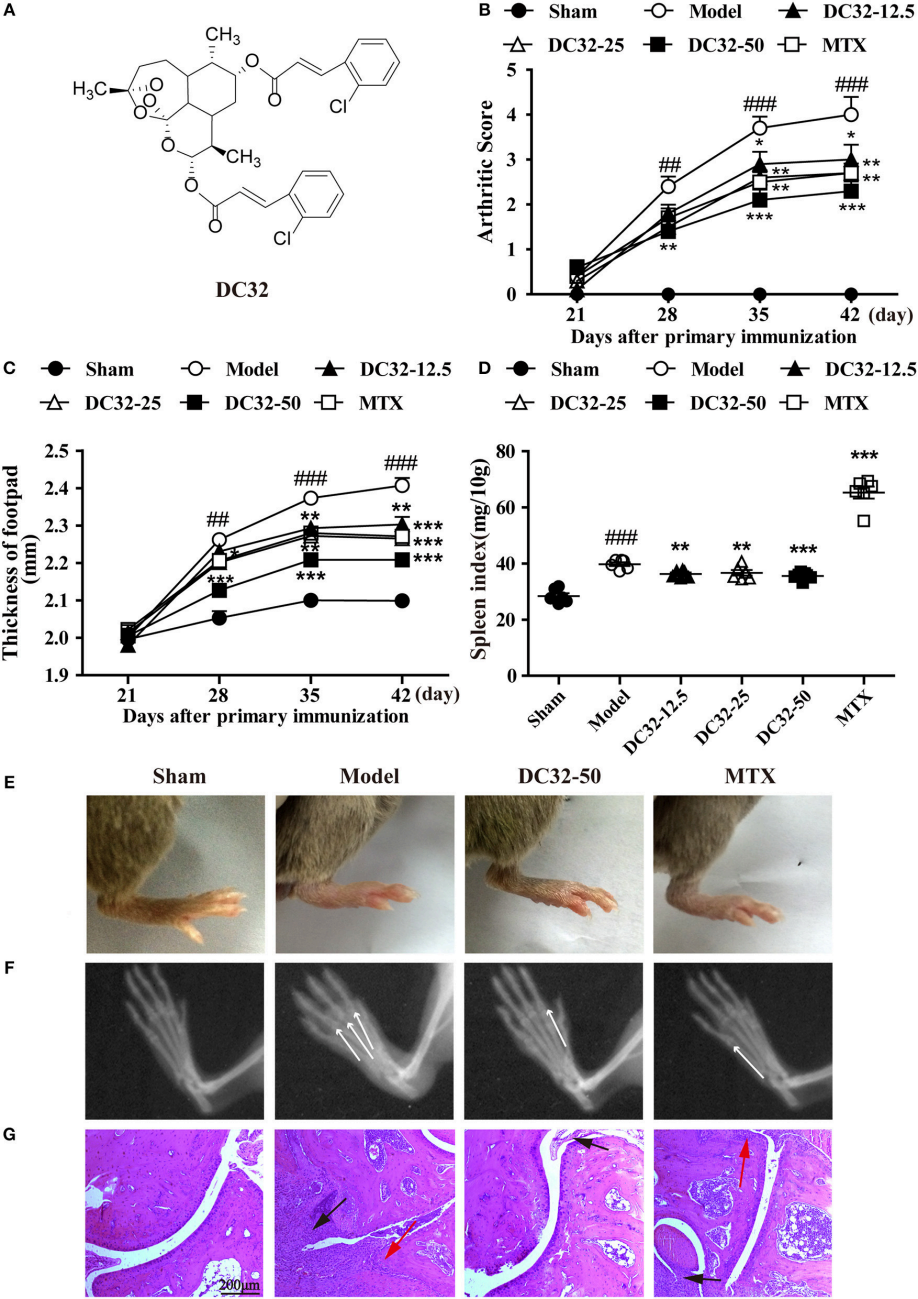
DC32 ameliorated CIA in DBA/1 mice. **(A)** Chemical structure of DC32. CIA mice were administered vehicle, DC32 (12.5, 25, 50 mg/kg) or MTX (2 mg/kg every 3 days) on day 26. **(B,C)** The arthritis score and footpad thickness were measured every week after secondary immunization. The footpad swelling was attenuated by administration of DC32 (*n* = 10). **(D)** The spleen index (ratio of spleen weight to body weight × 10) was calculated after the mice were sacrificed and administration of DC32 reduced the spleen index increased by CIA (*n* = 6). **(E–G)** Representative photographs of hind paws, radiological changes and HE-stained sections of ankle joints. The white arrow indicated the bone destruction in digital joints. The red arrow indicated chondrocytes and the black arrow indicated hyperplasia of connective tissues. DC32 attenuated the footpad swelling, articular destruction and degradation, synovial proliferation and inflammatory cell infiltration in CIA mice. The results are expressed as the mean ± SEM and analyzed by one-way analysis of variance (ANOVA) and two-way ANOVA followed by Bonferroni's *post-hoc* tests, ^##^*P* < 0.01, ^###^*P* < 0.001 vs. the Sham group; *P < 0.05, **P < 0.01, ***P < 0.001 vs. the Model group.

In our previous study, a series of dihydroartemisinin-cinnamic acid ester derivatives were synthesized by esterifying 9α-hydroxyl-dihydroartemisinin (30). Various combinations of dihydroartemisinin and cinnamic acids have been discovered with higher immunosuppressive abilities than those of either compound alone in the lymphocyte proliferation inhibition assay. DC32 (Figure 1A) was the most efficient among these compounds and was superior to artemisinin and artesunate in inhibiting lipopolysaccharide (LPS)- or concanavalin A (ConA)-induced lymphocyte proliferation. These results suggested that DC32 has the potential to treat autoimmune diseases, including RA (Figure S1A). This study evaluated the effects of DC32 on the CIA model and was expected to illuminate the autophagy-related Nrf2/HO-1 activation mechanisms of DC32.

## Materials and methods

### Animals

Pathogen-free male DBA/1 mice were purchased from Beijing Weitong Lihua Experimental Animal Co. Ltd. (Beijing, China) (SCXK2012-0001). The mice were housed in a laminar flow cabinet with a 12 h light/dark cycle and maintained on specific pathogen-free (SPF) laboratory chow and water *ad libitum*.

### Induction of CIA

Preparation of type II collagen (CII) emulsion: chicken CII was dissolved to a concentration of 2 mg/mL in 0.1 μM acetic acid overnight at 4°C and mixed with complete Freund's adjuvant (C9301, Sigma, USA). Male DBA/1 mice (6–8 weeks old) were immunized with 100 μg of chicken CII (100 μL). The day of the first immunization was defined as day 0. The mice then received a booster with an equal amount of chicken CII emulsified in Freund's incomplete adjuvant on day 21 (31, 32). Mice with CIA were divided into six groups (*n* = 10 per group): a sham group, a model group, three DC32-treated groups (12.5, 25, 50 mg/kg/d), and a methotrexate (MTX) group (2 mg/kg/ every 3 days). From day 26 to 46, three doses of DC32 and MTX (dissolved in corn oil) or vehicles (corn oil) were administrated by oral gavage to the CIA mice. The sham group was also injected with chloral hydrate on day 0 and administrated with vehicle (corn oil) from day 26 to day 46.

From day 21 after the first immunization, clinical arthritis scores were determined every week using a scoring system of 0–4 for each limb: 0, normal; 1, definite redness and swelling of the ankle or one digit; 2, two joints involved; 3, more than two joints involved; and 4, severe arthritis of the entire paw and all digits. The footpad thicknesses were also measured every week. On day 46, the mice were killed, and joint or paw tissues were harvested for hematoxylin and eosin staining (33) and protein extraction. Then, the spleens were removed and weighed to evaluate splenomegaly. The spleen index (ratio of spleen weight to body weight × 10) was calculated.

### Preparation of serum and protein samples

Blood was collected from the retro-orbital venous plexus. After standing for 30 min at room temperature (RT), the samples were centrifuged at 5,000 rpm for 15 min. We evaluated the Nrf2 protein expression by western blotting of hind paw homogenates (34–36). Briefly, the mice were sacrificed, and the ankle joint was cut. Frozen hind paws were homogenized in liquid N_2_. Then, the homogenate was suspended in 1 mL of ice-cold RIPA lysis buffer (Beyotime Biotechnology, China) with 10 μL of PMSF for 30 min for complete lysis. After centrifugation at 15,000 rpm for 10 min at 4°C, the supernatants were divided into aliquots and transferred to new tubes. All extraction procedures were performed on ice. Protein concentrations were then determined using a BCA protein assay kit (Thermo, USA).

### ELISA assay

The levels of HO-1 (ab204524, Abcam, UK), IgG (70-EK2712, Multisciences, China), and IgE (70-EK2752, Multisciences, China) in the serum were determined by ELISA kits.

### Western blot analysis

Antibodies: antibodies against Nrf2, Keap1, lamin B, Akt, p-Akt, mTOR, p-mTOR, JNK, p-JNK, ERK, p-ERK, p38, and p-p38 were purchased from CST (USA); antibodies against LC3, HO-1, and p62 were purchased from Abcam (UK); and anti-GAPDH antibody was purchased from Sigma (USA). Proteins were resolved by 10% SDS–polyacrylamide gel electrophoresis and transferred to a PVDF membrane (0.22 μm, Merck Millipore). The membranes were treated with 5% BSA-Tris buffered saline (TBS) containing 0.1% Tween 20 for 2 h to block non-specific binding, rinsed, and incubated with primary antibodies diluted in 5% w/v non-fat dry milk (or BSA when indicated by the instructions), 1 × TBS, and 0.1% Tween 20 at 4°C with gentle shaking overnight. Signals were detected with HRP-conjugated anti-rabbit IgG using an enhanced chemiluminescence system (Bio-Rad, USA) (26).

### Preparation of CIA synovial fibroblasts and cell culture

The synovium from CIA mice was removed and incubated for 1 h with 80 rpm shaking and 1 mg/mL collagenase (Sigma–Aldrich) at 37°C. The cells were then washed and cultured in DMEM-10% FBS. The fibroblast-like synoviocytes (FLSs) were used after 3–5 passages. NIH-3T3 cells were cultured in DMEM-10% FBS with Penicillin-Streptomycin.

### Preparation of nuclear and cytoplasmic samples

NIH-3T3 cells were seeded in 6-well plates at a density of 1 × 10^5^ cells/well overnight. LPS was added to the cells to generate a representative model of inflammation. After treatment with different concentrations of DC32 and LPS for 24 h, the cells were collected for protein extraction. The effect of DC32 on cell viability was determined by the MTT assay. For the detection of nuclear Nrf2 and Keap1 expression, nuclear and cytoplasmic fractions were separated by a nuclear and cytoplasmic protein extraction kit (Beyotime Biotechnology, China), according to the manufacturer's instructions. All steps were carried out on ice.

### Autophagy and proteasome inhibitor on NIH-3T3 cells

To confirm the role of autophagy in the regulation of Nrf2 and Keap1, we used the autophagy inhibitors 3-methyladenine (3-MA) and chloroquine (CQ) and the proteasome inhibitor MG-132 to block the degradation of Keap1 in NIH-3T3 cells. Cells were seeded in 6-well plates and pretreated with or without 5 mM 3-MA, 10 μM CQ, or 1 μM MG-132 before stimulation by LPS for 2 h. Then, DC32 (3 μM) was added to the corresponding wells.

### mRNA isolation and reverse transcription

The mRNA extraction kit (TransZol Up, ET111, Transgen, China) and reverse transcription kit (HiScript II Reverse Transcriptase, R223-01, Vazyme, China) were used according to the manufacturer's instructions.

### Quantitative real-time PCR (qPCR)

qPCR was performed according to the manufacturer's instruction (AQ131, Transgen, China). All values were expressed relative to the expression of GAPDH.

The following primers were used:

F(Nrf2):TAGATGACCATGAGTCGCTTG

R(Nrf2):GCCAAACTTGCTCCATGTCC;

F(HO-1): CCGCCTTCCTGCTCAACAT

R(HO-1):GCCACATTGGACAGAGTTCAC;

F(Keap1): CCGCAGAATGTTACTATCCAGAG

R(Keap1): CGCTCCACACTGTTCAACTG;

F(p62): ATGTGGAACATGGAGGGAAGA

R(p62): GGAGTTCACCTGTAGATGGGT;

F(GA): TGATGGGTGTGAACCACGAG

R(GA): GCCCTTCCACAATGCCAAAG;

### Immunofluorescence assay

The translocation of Nrf2 was determined using an immunofluorescence assay (IF). The FLSs (5,000 cells/well in a 24-well plate) were seeded on glass coverslips overnight. Then, 1 mg/mL LPS and DC32 (1, 3, 10 μM) were added to the corresponding groups and cultured for 24 h. The cells were then fixed with 4% paraformaldehyde, washed and incubated with blocking buffer for 2 h at RT. The cells were incubated with the Nrf2 antibody (1:100) for 16–19 h at RT and then washed and incubated with biotin-conjugated anti-rabbit IgG (1:300) for 2 h. The cells were washed again and incubated with DAPI for 5 min. The coverslips were fixed on slides with 50% glycerin.

### Gene knockdown in NIH-3T3 cells

Predesigned small interfering RNA (siRNA) for p62 (sense: GGACCCAUCUACAGGUGAAT, antisense: UUCACCUGUAGAUGGGUCCTT') and Nrf2 (sense: GCAAGUUUGGCAGGAGCU-ATT, antisense: UAGCUCCUGCCAAACUUGCTT) and negative-control siRNA were purchased (TranSheepBio, China). Transfection mixes were prepared using Namipo. Cells at 30% confluence were transfected and then cultured for 24 h before treatment with DC32 or LPS.

### Statistical analysis

SPSS (version 15.0) and GraphPad Prism software (Version 6.0) were used to conduct the statistical analyses. After a Kolmogorov-Smirnov non-parametric test for normality, data were statistically evaluated by one-way analysis of variance (ANOVA) and two-way ANOVA followed by Bonferroni's *post-hoc* tests. *P* < 0.05 was considered significant. Data are presented as the mean ± SEM.

## Results

### DC32 ameliorated CIA in DBA/1 mice

DC32 and MTX were given to mice with CIA from day 26 to 46, and the clinical arthritis scores and footpad thickness were measured every week to evaluate the therapeutic effect of DC32. DC32 significantly reduced the clinical arthritis scores and footpad thickness (Figures 1B,C) without influencing the body weight (Figure S1F). The spleen index was measured after the mice were sacrificed. The results suggested that DC32 attenuated the splenomegaly caused by immunologic abnormalities (Figure 1D), the footpad swelling (Figure 1E) and the inflammation in CIA mice. Radiographs of the hind paws in the model group showed articular destruction and degradation of the bone matrices. However, bone destruction was obviously attenuated in the DC32-treated mice (Figure 1F). The HE sections revealed that the ankle joints in CIA mice were seriously damaged by synovial proliferation and inflammatory cell infiltration. DC32 treatment led to a remarkable improvement in inflammation and joint destruction (Figure 1G).

### DC32 reduced inflammation and RFs *in vivo*

**Figure 2:**
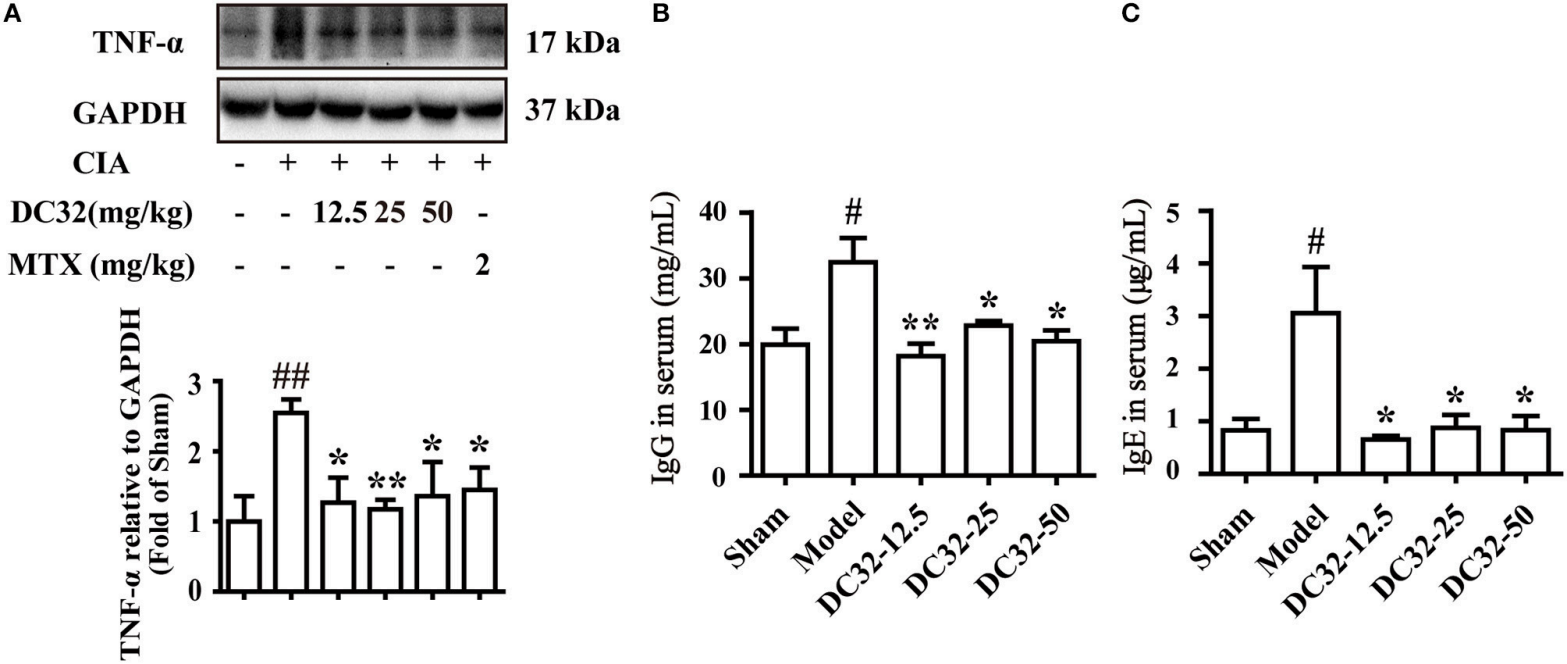
DC32 reduced inflammation and rheumatoid factors (RFs) *in vivo*. **(A)** The TNF-α in hind paw homogenates was analyzed by western blotting, and the TNF-α level was decreased by administration of DC32 (12.5, 25, 50 mg/kg) and MTX (2 mg/kg) (*n* = 3–4). **(B,C)** The concentrations of RFs (IgG and IgE) in serum were measured by ELISA, and DC32 administration decreased the RF levels in serum (*n* = 3). The results are expressed as the mean ± SEM and analyzed by one-way analysis of variance (ANOVA) and two-way ANOVA followed by Bonferroni's *post-hoc* tests, ^#^*P* < 0.05, ^##^*P* < 0.01 vs. the Sham group; *P < 0.05, **P < 0.01 vs. the Model group.

TNF-α was significantly reduced in all treatment groups, demonstrating the anti-inflammatory activity (Figure 2A). The rheumatoid factors (RFs) IgG and IgE were significantly upregulated in the model group, indicating that humoral immunity was activated. IgG and IgE were remarkably decreased in the DC32 groups in a dose-independent manner (Figures 2B,C).

### DC32 activated the Nrf2/HO-1 pathway in CIA mice and the LPS-induced inflammatory model in NIH-3T3 cells

**Figure 3:**
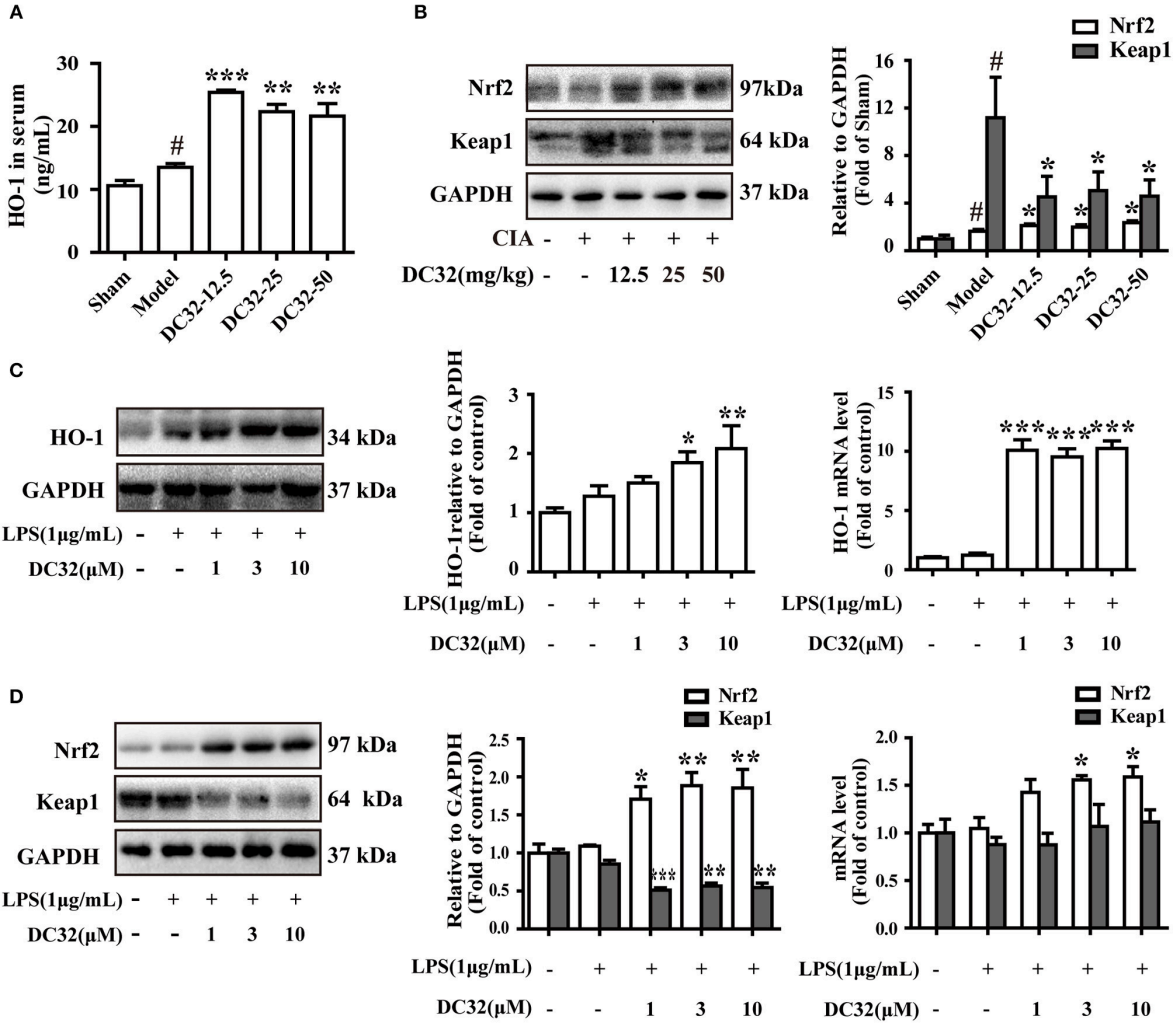
DC32 activated the Nrf2/HO-1 pathway in CIA mice and the LPS-induced inflammatory model in NIH-3T3 cells. **(A,B)** The concentration of HO-1 in serum was measured by ELISA (*n* = 3). The Nrf2 and Keap1 in hind paw homogenates were analyzed by western blotting. DC32 (12.5, 25, 50 mg/kg) significantly increased the level of HO-1 and Nrf2 and reduced the expression of Keap1 in the hind paws (*n* = 3–4). The results are expressed as the mean ± SEM of three experiments, ^#^*P* < 0.05 *vs*. the Sham group; *P < 0.05, **P < 0.01, ***P < 0.001 *vs*. the Model group. **(C,D)** The Nrf2, Keap1 and HO-1 protein levels and mRNA expression (24 h) were measured by western blotting (*n* = 4) and qPCR (*n* = 5) *in vitro*. DC32 increased the expression and transcription of Nrf2 and HO-1, and reduced the level of Keap1 without influencing its transcription. The results are expressed as the mean ± SEM and analyzed by one-way analysis of variance (ANOVA) and two-way ANOVA followed by Bonferroni's *post-hoc* tests, *P < 0.05, **P < 0.01, ***P < 0.001 vs. the LPS group.

The Nrf2/HO-1 antioxidant pathway could be activated by inflammation and ROS (37). Consistent with the results in RA patients, the expression levels of HO-1 and Nrf2 in CIA mice were slightly increased (7, 19). We found that DC32 could further activate the Nrf2/HO-1 antioxidant pathway and relieve inflammatory symptoms in mice with CIA. The HO-1 concentration in serum was measured by ELISA (Figure 3A). The HO-1 level in the DC32 group obviously increased. The Nrf2 level was upregulated significantly in the DC32 groups in a slightly dose-dependent manner (Figure 3B). The above results have proven that Nrf2/HO-1 may play an important role the DC32-amelioration of CIA, which was in harmony with the previous view that Nrf2/HO-1 is a therapeutic target in RA. We evaluated the effects of DC32 on Nrf2/HO-1 in NIH-3T3 cells and the cytotoxicity was analyzed (Figure S1B). When DC32 (1, 3, 10 μM) was given to NIH-3T3 cells for 24 h, both the protein and mRNA levels of HO-1 were significantly upregulated (Figure 3C). Moreover, Nrf2 expression was also increased, whereas the level of Keap1 was oppositely regulated. To clarify the mechanism underlying this effect, we measured the mRNA levels of Nrf2 and Keap1 at 24 h (Figure 3D). The results showed that DC32 activated Nrf2/HO-1 in CIA mice. The transcription of Nrf2 was increased, and the transcription of HO-1 was 10 times higher than that in the control NIH-3T3 cells. However, the mRNA level of Keap1 was not different between the groups. Therefore, the decrease in Keap1 was not caused by inhibiting its transcription.

### DC32 facilitated the nuclear translocation of Nrf2

**Figure 4:**
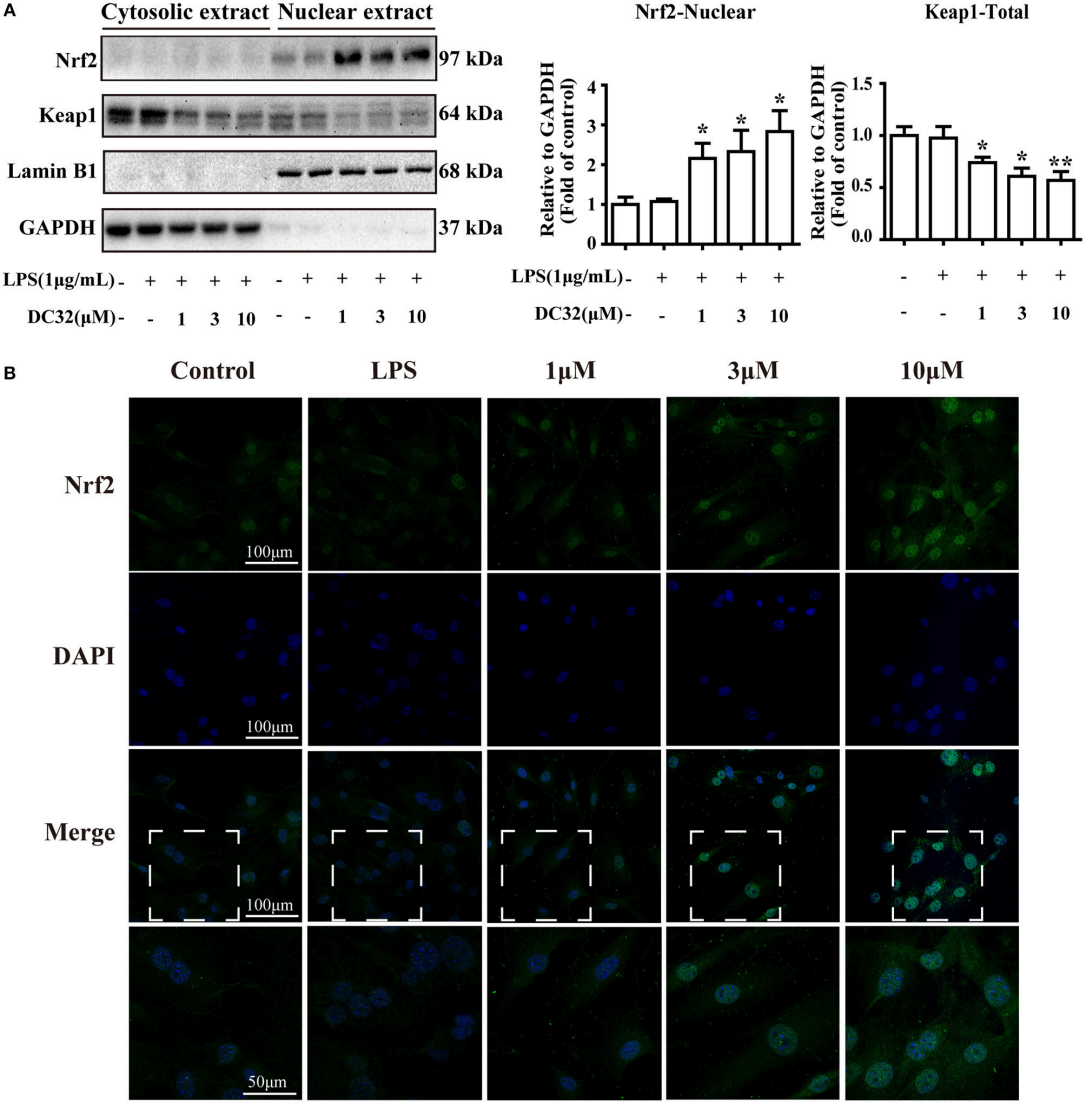
DC32 facilitated the nuclear translocation of Nrf2. Cells were treated with LPS (1 mg/mL) and DC32 (1, 3, 10 μM) for 24 h. **(A)** The nuclear and cytoplasmic levels of Nrf2 and Keap1 were examined by western blot analysis (*n* = 4). Nrf2 level in nuclear was increased by treating with DC32. **(B)** FLSs were fixed and subjected to immunofluorescence (IF) staining with Nrf2 antibody (green), and the nucleus was stained with DAPI (blue). Increase of nuclear level of Nrf2 was observed after treating with DC32. The images in fourth row are magnified images of the white boxes in the third row. DC32 increased the nuclear translocation of Nrf2. The results are expressed as the mean ± SEM and analyzed by one-way analysis of variance (ANOVA) and two-way ANOVA followed by Bonferroni's *post-hoc* tests, *P < 0.05, **P < 0.01 vs. the LPS group.

Nrf2 translocates into the nucleus and activates expression of its related genes. To determine whether DC32 increased HO-1 expression by facilitating Nrf2 translocation, nuclear, and cytoplasmic protein extraction and IF were carried out. The western blot results showed that DC32 significantly increased Nrf2 nuclear translocation and reduced Keap1 levels in the cytoplasm (Figure 4A). Nrf2 nuclear translocation was further confirmed by an immunofluorescence (IF) assay (Figure 4B). In the control group, Nrf2 protein was mainly localized in the cytoplasm, but a low density was observed in the nucleus. After treatment with DC32, Nrf2 was exclusively found in the nucleus, which was consistent with the nuclear and cytoplasmic protein separation results. Additionally, our results further suggested that 10 μM DC32 resulted in a significant increase in the nuclear expression of Nrf2 and a concomitant decrease in its cytoplasmic levels.

### DC32 induced Keap1 degradation by promoting p62 in CIA mice and NIH-3T3 cells

**Figure 5:**
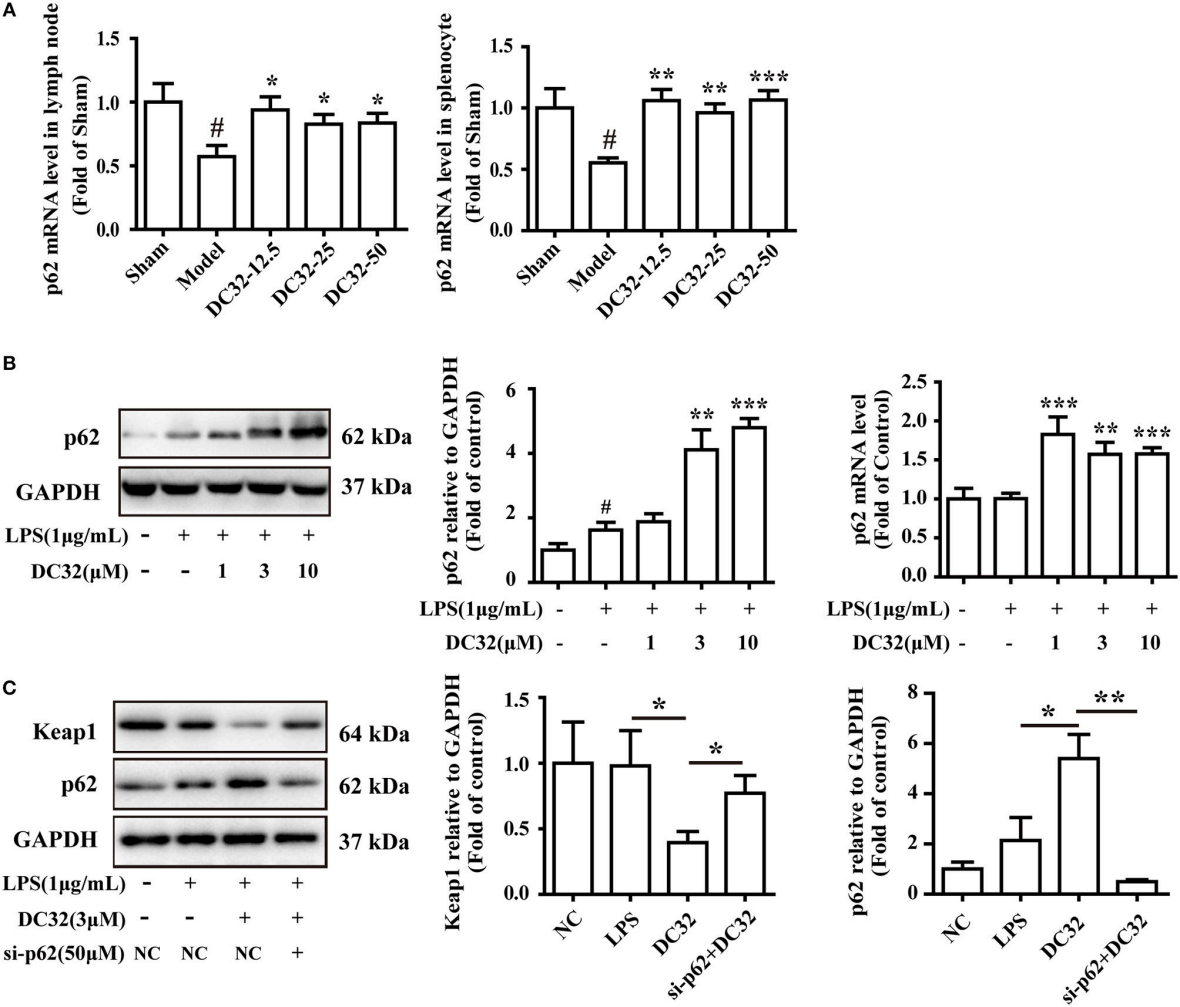
DC32 induced Keap1 degradation by promoting p62 expression in CIA mice and NIH-3T3 cells. **(A)** The relative levels of p62 mRNA expression in the lymph nodes and splenocytes were measured by qPCR (*n* = 4). DC32 (12.5, 25, 50 mg/kg) increased the mRNA expression of p62. The results are expressed as the mean ± SEM of three experiments, ^#^*P* < 0.05 vs. the Sham group, *P < 0.05, **P < 0.01, ***P < 0.001 vs. the Model group. **(B)** The effect of DC32 on p62 expression and transcription in NIH-3T3 cells was determined by western blotting (*n* = 4) and qPCR (*n* = 5). The p62 expression and transcription in NIH-3T3 cells were increased by DC32. **(C)** Treatment with 3 μM DC32 resulted in a decrease in Keap1, which was relieved by p62 siRNA (*n* = 4). The results are expressed as the mean ± SEM and analyzed by one-way analysis of variance (ANOVA) and two-way ANOVA followed by Bonferroni's *post-hoc* tests, *P < 0.05, **P < 0.01.

To further evaluate the relationship between p62 and Keap1 degradation, the effect of DC32 on p62 expression and transcription was determined by western blotting and qPCR. DC32 significantly increased the p62 mRNA levels in CIA mice (Figure 5A), as well as the p62 expression and transcription in NIH-3T3 cells (Figure 5B). Moreover, opposite trends for p62 and Keap1 were observed in each group, which hinted that DC32 regulated Keap1 in a p62-dependent manner. To confirm the role of p62 in Keap1 degradation, predesigned siRNA for p62 was transfected into NIH-3T3 cells. The siRNA-mediated knockdown of p62 (Figure S1C) increased the Keap1 protein level, which indicated that the DC32-induced degradation of Keap1 was mediated by p62 (Figure 5C). Our results indicated that DC32 could enhance the transcription and expression of p62 and facilitate the degradation of Keap1. The degradation of Keap1 could be inhibited by the siRNA interference of p62, and the effect of DC32 on Keap1 could be reversed by the siRNA for p62. This result suggested that the degradation of Keap1 induced by DC32 was mediated by p62, which blocked the proteasomal degradation of Nrf2.

### DC32 inhibited ERK phosphorylation and induced autophagy

**Figure 6:**
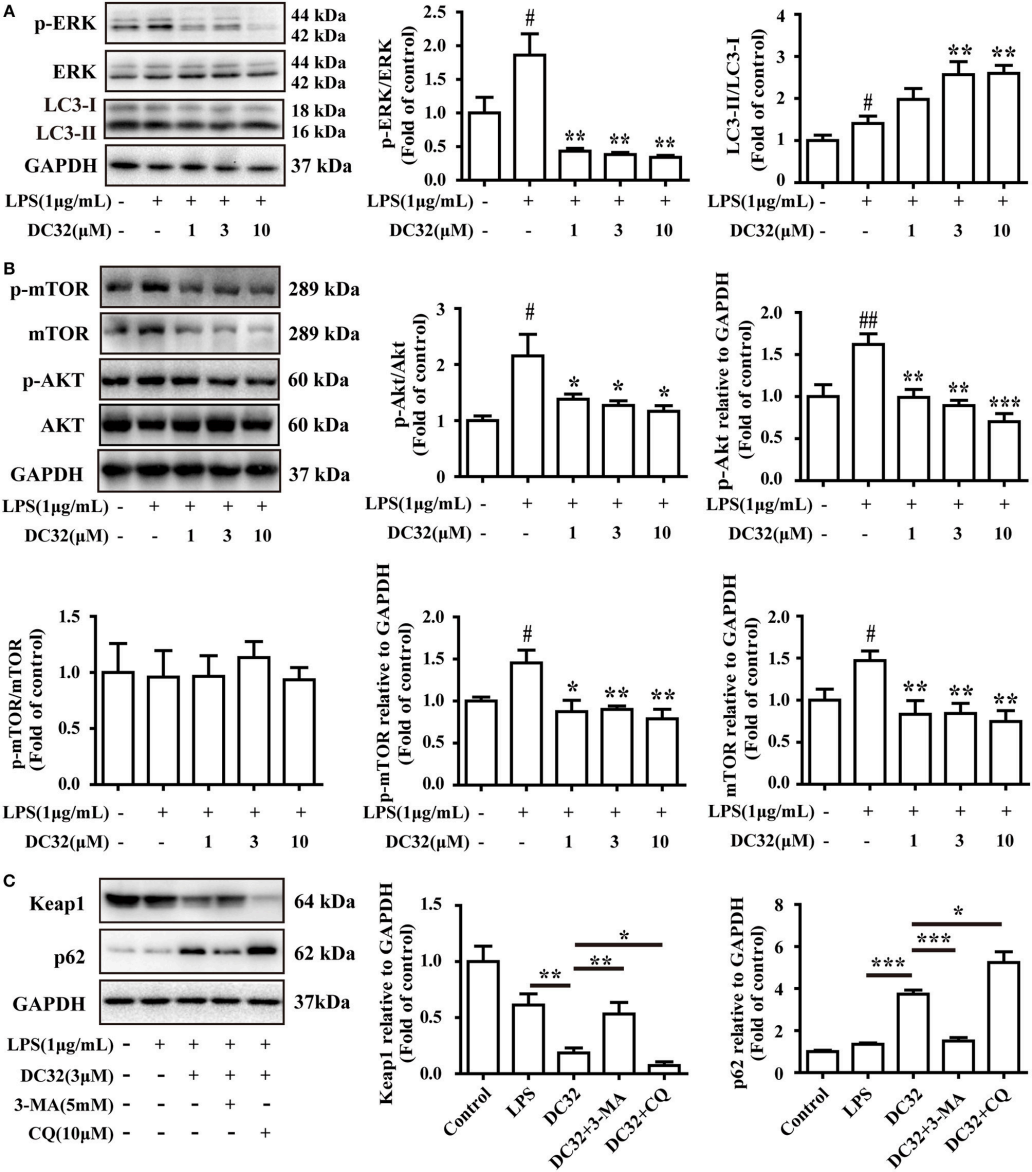
DC32 inhibited ERK phosphorylation and induced autophagy. **(A)** Phosphorylation of ERK was inhibited and the ratio of LC3II/LC3I was increased by treating with DC32 (*n* = 4); **(B)** phosphorylation of Akt and expression of mTOR was inhibited by treating with DC32 (*n* = 4); **(C)** Keap1 and p62 expression after pretreatment with 5 mM 3-MA and 10 μM CQ. Keap1 expression was decreased significantly with 3 μM DC32, and partly abolished by 3-MA (*n* = 4). The results are expressed as the mean ± SEM and analyzed by one-way analysis of variance (ANOVA) and two-way ANOVA followed by Bonferroni's *post-hoc* tests, ^#^*P* < 0.05 vs. the control group; *P < 0.05, **P < 0.01, ***P < 0.001 *vs*. the LPS group in **(A)**; *P < 0.05, **P < 0.01 in **(B,C)**.

Selective autophagy is closely connected to p62 and Keap1. Autophagy is reported to be regulated by the MAPK pathway. Thus, the phosphorylation of JNK, ERK, and p38 was analyzed by western blotting. The results showed that DC32 significantly decreased ERK phosphorylation (Figure 6A) but had no influence on JNK and p38 phosphorylation (Figure S1D). Moreover, DC32 dramatically enhanced autophagy by increasing LC3II/LC3I (Figure 6A). These results revealed that DC32 remarkably induced autophagy by inhibiting ERK phosphorylation. DC32 significantly inhibited the phosphorylation of Akt. However, DC32 reduced the phosphorylation of mTOR by inhibiting its expression (Figure 6B). The autophagy inhibitors 3-MA and CQ were administered to NIH-3T3 cells, and the change in protein levels was determined by western blotting. Keap1 expression was decreased significantly with 3 μM DC32, and this phenomenon was partly abolished by 3-MA. MG-132, a proteasome inhibitor, was given to NIH-3T3 to determine whether the proteasome was involved in this Keap1 degradation. Both CQ and MG-132 caused aggravated degradation of Keap1 and accumulation of p62 Figure 6C, Figure S1E).

### Nrf2 is upstream of p62 in the DC32-induced Nrf2-Keap1-p62 feedback loop

**Figure 7:**
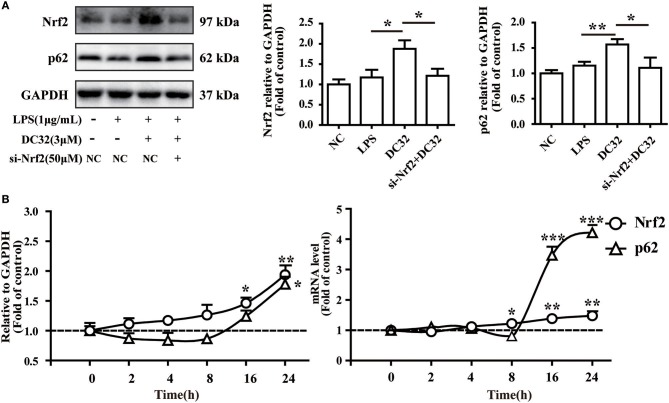
Nrf2 is upstream of p62 in the DC32-induced Nrf2-Keap1-p62 feedback loop. **(A)** Treatment with 3 μM DC32 resulted in increases in Nrf2 and p62, which were relieved by Nrf2 siRNA (*n* = 4). The results are expressed as the mean ± SEM of three experiments, *P < 0.05, **P < 0.01. **(B)** The expression and transcription of Nrf2 and p62 at 2, 4, 8, 16, and 24 h were measured by western blotting and qPCR (*n* = 4). The expression and transcription of Nrf2 were consistently increased with time, whereas the expression and transcription of p62 first decreased and then increased at 16 h. The results are expressed as the mean ± SEM and analyzed by one-way analysis of variance (ANOVA) and two-way ANOVA followed by Bonferroni's *post-hoc* tests, *P < 0.05, **P < 0.01, ***P < 0.001 *vs*. the LPS group.

Nrf2 regulates p62 transcriptionally and may further enhance the accumulation of Nrf2. To verify the role of Nrf2 in the expression of p62, siRNA for Nrf2 was transfected into NIH-3T3 cells. The change in the protein level of p62 was determined by western blotting. The results showed that the increase in p62 induced by DC32 was attenuated by Nrf2 siRNA (Figure 7A, Figure S1C). In addition, the effects of DC32 on the expression and transcription of Nrf2 and p62 at 2, 4, 8, 16, and 24 h were measured by western blotting and qPCR (Figure 7B). The expression and transcription of Nrf2 were consistently increased with time, whereas the expression and transcription of p62 first decreased and then increased at 16 h. These results confirmed that Nrf2 is upstream of this DC32-induced Nrf2-Keap1-p62 feedback loop.

## Discussion

**Figure 8:**
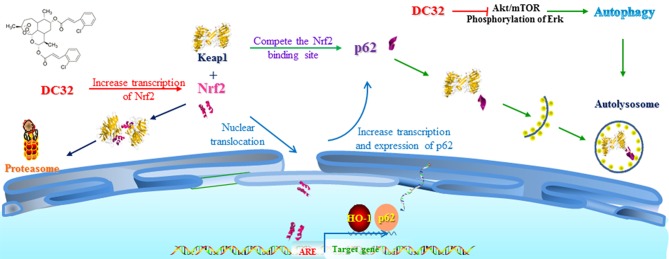
Mechanisms of DC32-induced Nrf2 activation. DC32 induced autophagy by inhibiting Akt/mTOR and ERK activation. DC32 also increased the mRNA level and nuclear translocation of Nrf2 at the same time and then promoted p62 transcription and expression. The degradation of Keap1 via the autolysosome was then facilitated by p62. The Nrf2-p62-Keap1 feedback loop was subsequently triggered and further amplified the activation of Nrf2/HO-1.

In this study, we revealed that DC32 ameliorated the footpad swelling, cartilage degradation, and inflammatory cell infiltration of joints downregulated the TNF-α level in footpads and the increased serum concentration of RFs (IgG and IgE) in mice with CIA. Further mechanistic exploration indicated that DC32 ameliorated CIA via the Nrf2-p62-Keap1 feedback loop by increasing the mRNA and protein levels of Nrf2 and enhancing the expression of p62 (Figure 8).

Activation of autophagy can be regarded as a therapeutic target for RA and related autoimmune diseases. Enhanced autophagy activity reduces the production of proinflammatory cytokines in RA FLSs (38), while heat shock proteins (HSPs) and caveolin-1 protect against autoimmune diseases in an autophagy-related way (39, 40). In our research, we found that DC32 activated autophagy and exerted anti-inflammatory effects. In addition, DC32 also restored the imbalance of the T lymphocyte subpopulation and ameliorated lymphocytic infiltration in CIA mice (41). These functions were probably regulated by autophagy- and Nrf2/HO-1-related pathways.

It has been reported that inhibiting the activation of Akt/mTOR and ERK could induce autophagy (42–44) and that the LC3II/I ratio is subsequently increased (45). Our results revealed that DC32 induced selective autophagy by inhibiting the activation of Akt/mTOR and ERK. Autophagy inhibitor 3-MA could attenuate the degradation of Keap1 induced by DC32 but not the autolysosome inhibitor CQ or the proteasome inhibitor MG-132 in NIH-3T3 cells stimulated with LPS. It has been reported that 3-MA can inhibit the activity of class III PI3Ks and that CQ blocks autophagic flux by affecting lysosomal acidification (46). CQ inhibits the function of autophagosome which leads to the accumulation of p62. This accumulation may enhance the isolation of Keap1 by p62. In addition, there is reported that CQ can activate Nrf2/HO-1 pathway (14). Therefore, we speculated that the accumulation of p62 and degradation of Keap1 induced by DC32 were controlled by PI3K-III. MG-132 could block the degradation of p62 through the proteasome, which may lead to extensive lysosomal degradation of Keap1. The results of 3-MA and p62 mRNA interfering with Keap1 in NIH-3T3 cells stimulated with LPS indicated that the accumulation of p62 further enhanced the autophagic degradation of Keap1 and suggested that DC32 may also induce autophagy by regulating class III PI3Ks (47–49). This study provided further evidence that autophagy plays a role in treating autoimmune diseases, and understanding the effects of autophagy on autoimmune diseases will contribute to the discovery of new therapeutic strategies.

In this study, Nrf2 mRNA interference prevented the DC32-induced upregulation of p62, proving that Nrf2 is upstream of the p62 regulation of DC32. DC32 augmented the Nrf2 level by increasing the transcription of Nrf2 and inhibiting the degradation of Nrf2, and the transcription of p62 was subsequently increased. However, we discovered that the expression of p62 did not increase immediately after the increase in Nrf2 caused by DC32 administration. This result might be because p62, as an autophagy substrate, could be degraded by autophagic flux. p62 was degraded along with Keap1 in autophagic flux, resulting in the observed delay in p62 upregulation.

The Nrf2-p62-Keap1 feedback loop is responsible for cellular homeostasis and is involved in a protective role against hepatotoxicity in human normal liver cells (50). We also found that DC32 has showed hepaprotective effect in this research (Figure S1G). Considering new strategies involving the Nrf2-p62-Keap1 feedback loop will shed light on the development of drugs for diseases associated with inflammation and immune dysfunction. However, there is little research on the role of the Nrf2-p62-Keap1 feedback loop in autoimmune diseases to date. The DC32-induced Nrf2-p62-Keap1 feedback loop further enhanced the activation of Nrf2/HO-1 and ameliorated autoimmune arthritis. This finding suggests new mechanisms for artemisinins in the treatment of RA and further supports the role of the Nrf2-p62-Keap1 feedback loop in autoimmune diseases. Current research speculates that this feedback loop will be disrupted by the degradation of p62 (17). As mentioned above, Nrf2 mRNA interference was discovered to increase the LC3II/I ratio further in our study. Therefore, we hypothesized that a negative feedback loop might exist between Nrf2 and autophagy, which merits further study to illuminate the complex relationship between Nrf2 and autophagy.

The antiarthritic effect of DC32 was evaluated in mice with CIA. Our findings provided pharmacological evidence supporting the potential of DC32 in RA therapy and illuminated the mechanisms of Nrf2/HO-1 activation: DC32 increased the transcription and nuclear translocation of Nrf2 and then facilitated the transcription of p62, which magnified the activation of Nrf2/HO-1 by an autophagy-dependent Nrf2-p62-Keap1 feedback loop (51). These findings provide a new mechanism for artemisinins in RA therapy, and DC32 could be regarded as a new antirheumatic candidate.

## Ethics statement

All animal care procedures and experiments in this study were carried out in accordance with the recommendations of National Institutes of Health Guide for the Care and Use of Laboratory Animals, and the protocols used were approved by the Animal Ethics Committee of China Pharmaceutical University.

## Author contributions

MF designed and carried out the experiments, analyzed the data, and wrote and discussed the manuscript. YL assisted with the experiments and revised the manuscript. CY synthesized and purified the DC32 used in this study. JL, XL, and BY reviewed the manuscript and supervised the project.

### Conflict of interest statement

The authors declare that the research was conducted in the absence of any commercial or financial relationships that could be construed as a potential conflict of interest.
