# Supplementary material for: DC32, a Dihydroartemisinin Derivative, Ameliorates Collagen-Induced Arthritis Through an Nrf2-p62-Keap1 Feedback Loop
Source: Front Immunol. 2018 Nov 27;9:2762. doi: 10.3389/fimmu.2018.02762 (PMC6277526; doi:10.3389/fimmu.2018.02762)
Supplement: Supplementary file 1 [file Data_Sheet_1.docx]

**SUPPLEMENTARY MATERIAL**

Figure S1 (A) Inhibition ratio of DC32, Art and Ast (3 μM) in LPS- and ConA-induced lymphocyte proliferation. (B) DC32 (1–10 µM) showed no significant cytotoxicity on NIH-3T3 cells. (C) p62 and Nrf2 siRNA interference efficiency. (D) DC32 (1, 3, 10 µM) didn’t significantly influence the phosphorylation of JNK and p38. (E) Keap1 and p62 expression after pretreatment with 3 µM MG-132, MG-132 caused aggravated degradation of Keap1 and accumulation of p62. (F) Body weights of each group on day 46. DC32 didn’t cause weight loss. (G) ALT and AST levels in serum. DC32 has showed hepaprotective effect.


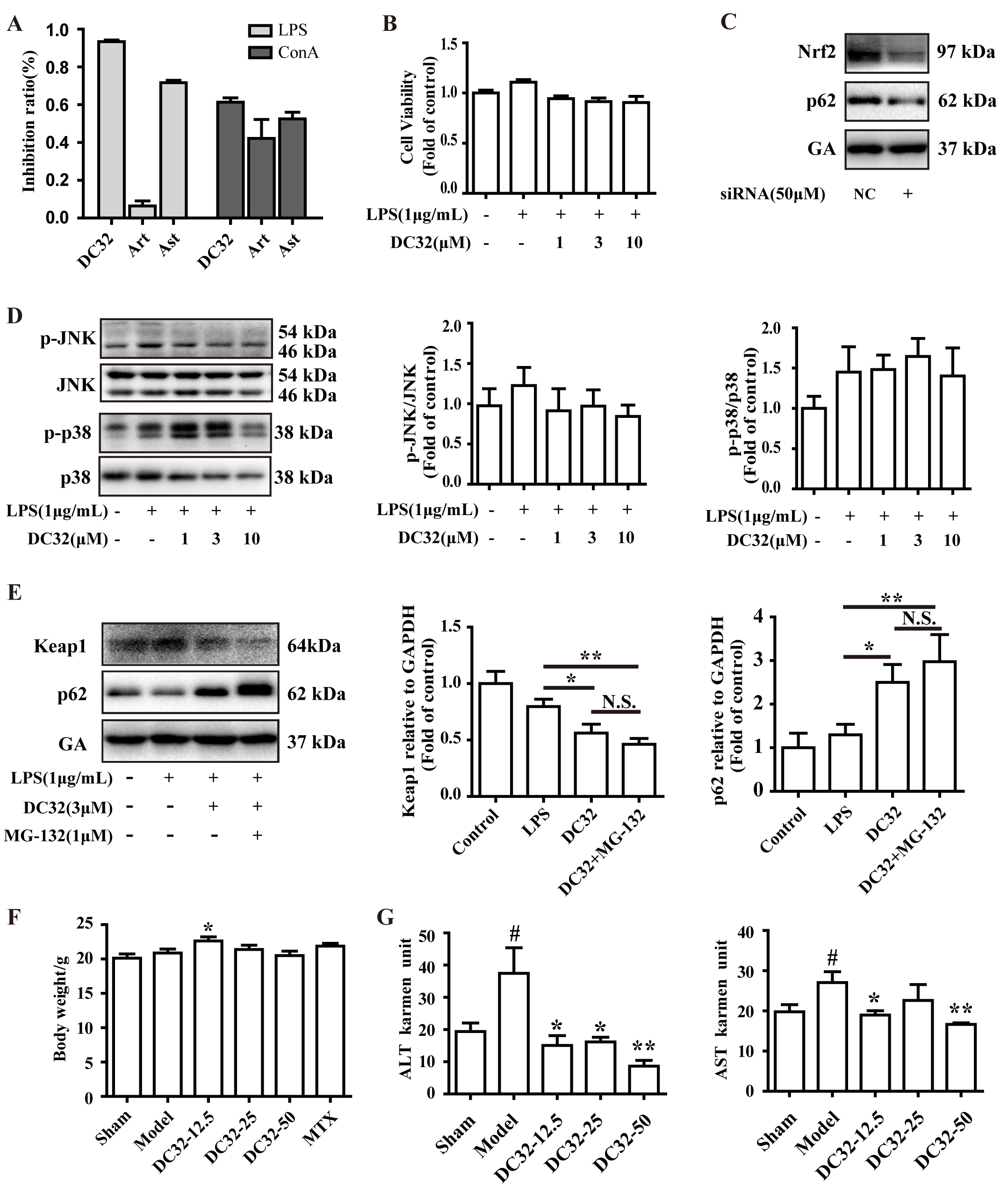


**Figure S1 (A) Inhibition ratio of DC32, Art and Ast (3 μM) in LPS- and ConA-induced lymphocyte proliferation. (B) DC32 (1–10 µM) showed no significant cytotoxicity on NIH-3T3 cells. (C) p62 and Nrf2 siRNA interference efficiency. (D) DC32 (1, 3, 10 µM) didn’t significantly influence the p**hosphorylation of JNK **and p38. (E)** Keap1 and p62 expression after pretreatment with 3 µM MG-132, MG-132 caused aggravated degradation of Keap1 and accumulation of p62. (F) Body weights of each group on day 46. DC32 didn’t cause weight loss. (G) ALT and AST levels in serum. DC32 has showed hepaprotective effect.
